# Evaluating the Increased Burden of Cardiorespiratory Illness Visits to Adult Emergency Departments During Flu and Bronchiolitis Outbreaks in the Pediatric Population: Retrospective Multicentric Time Series Analysis

**DOI:** 10.2196/25532

**Published:** 2022-03-10

**Authors:** Benoit Morel, Guillaume Bouleux, Alain Viallon, Maxime Maignan, Luc Provoost, Jean-Christophe Bernadac, Sarah Devidal, Sylvie Pillet, Aymeric Cantais, Olivier Mory

**Affiliations:** 1 Department of Pediatric Emergency University Hospital of Saint Etienne Saint Etienne France; 2 Décision et Information pour les Systèmes de Production EA4570 University of Lyon Villeurbanne France; 3 Emergency Department and Intensive Care Unit University Hospital Saint Etienne France; 4 Emergency Department and Mobile Intensive Care Unit University Grenoble Alpes La Tronche France; 5 Department of Pediatric Emergency Hospital University Grenoble France; 6 Department of Information Technology Hospital University Saint Etienne France; 7 Laboratory of Infectious Agents and Hygiene University Hospital of Saint Etienne Saint Etienne France; 8 Groupe sur l'Immunité des Muqueuses et Agents Pathogènes EA 3064 Saint Etienne France

**Keywords:** respiratory infections, emergency departments, flu outbreak, bronchiolitis outbreak, cardiorespiratory illness, time series analysis, influenza, bronchiolitis, outbreak, pediatrics

## Abstract

**Background:**

Cardiorespiratory decompensation (CRD) visits have a profound effect on adult emergency departments (EDs). Respiratory pathogens like respiratory syncytial virus (RSV) and influenza virus are common reasons for increased activity in pediatric EDs and are associated with CRD in the adult population. Given the seasonal aspects of such challenging pathology, it would be advantageous to predict their variations.

**Objective:**

The goal of this study was to evaluate the increased burden of CRD in adult EDs during flu and bronchiolitis outbreaks in the pediatric population.

**Methods:**

An ecological study was conducted, based on admissions to the adult ED of the Centre Hospitalier Universitaire (CHU) of Grenoble and Saint Etienne from June 29, 2015 to March 22, 2020. The outbreak periods for bronchiolitis and flu in the pediatric population were defined with a decision-making support tool, PREDAFLU, used in the pediatric ED. A Kruskal-Wallis variance analysis and a Spearman monotone dependency were performed in order to study the relationship between the number of adult ED admissions for the International Classification of Diseases (ICD)-10 codes related to cardiorespiratory diagnoses and the presence of an epidemic outbreak as defined with PREDAFLU.

**Results:**

The increase in visits to the adult ED for CRD and the bronchiolitis and flu outbreaks had a similar distribution pattern (CHU Saint Etienne: *χ*^2^_3_=102.7, *P*<.001; CHU Grenoble: *χ*^2^_3_=126.67, *P*<.001) and were quite dependent in both hospital settings (CHU Saint Etienne: Spearman *ρ*=0.64; CHU Grenoble: Spearman *ρ*=0.71). The increase in ED occupancy for these pathologies was also significantly related to the pediatric respiratory infection outbreaks. These 2 criteria gave an idea of the increased workload in the ED due to CRD during the bronchiolitis and flu outbreaks in the pediatric population.

**Conclusions:**

This study established that CRD visits and bed occupancy for adult EDs were significantly increased during bronchiolitis and pediatric influenza outbreaks. Therefore, a prediction tool for these outbreaks such as PREDAFLU can be used to provide early warnings of increased activity in adult EDs for CRD visits.

## Introduction

Respiratory infections have a strong impact on the number of visits in pediatric emergency departments (EDs) during the epidemic periods of flu and bronchiolitis [1]. Seasonal respiratory pathogen activity is also linked to increased use of health care services across patients of all ages [2,3]. In the pediatric population, a diagnosis of a respiratory infection is easy to make. The broad use of rapid flu tests in the pediatric ED allows an easy and precise diagnosis of flu infections. The symptoms of bronchiolitis are well identified and lead to an easy diagnosis. In the adult population, increased activity in the adult ED during respiratory pathogen activity is related to an excess of respiratory complaints [4] but also with more diverse causes, mostly due to patient comorbidities [5].

In the pediatric ED, influenza activity and respiratory syncytial virus (RSV) circulation are the main cause of seasonal overload. The PREDAFLU application was developed for the Centre Hospitalier Universitaire (CHU) Grenoble and Saint Etienne in order to anticipate the overload of activity due to respiratory infections in the pediatric population. This application allows a real-time analysis of pediatric emergency admissions in order to provide an early warning of increased activity related to influenza and bronchiolitis [6,7]. This surveillance helps determine the level and trend of respiratory infections. When an outbreak of respiratory infection is identified, this information is used to trigger additional resources in the hospital: physicians, nurses, beds. This knowledge allows pediatric EDs to be more agile and better prepared for the additional load. For the adult population, the increase in activity, morbidity, and mortality due to respiratory infections represents a significant burden for health services [8-10]. Although an accurate tool such as PREDAFLU is available in pediatrics, it is a challenge for the adult population [11,12].

This challenge is indeed illustrated in Multimedia Appendix 1. Taking all the pathogen codes associated with respiratory infections, the daily patient flow is displayed. It is very clear on this figure that the number of patients evolves in a quasiperiodic way on the one hand but with several periodicities and an extremely important variability. We also notice that infections are diagnosed during the summer months, which has no correlation with a viral presence of influenza or RSV and does not cause any noticeable congestion in the EDs. In order to have the same conditions of preventive detection as the conditions of exercise in the PREDAFLU tool, we used the set of early detectors in PREDAFLU on the adult patient flow. As we could expect, the detectors detected the start of an epidemic in the months of June, July, or December. Even if we suppress the June or July alarm, the detection obtained is much later than the detection proposed by the pediatric patient analysis. This indicates the difficulty of giving an early, daily, and reliable alarm for the increase in adults.

Given that the pathogens, RSV and influenza virus, are common for both populations, using PREDAFLU as an early detector of increased activity for both the pediatric and adult populations would add value for the ED.

The primary objective of this study was to determine if the increase in CRD admissions to the adult ED during respiratory infection outbreaks in the pediatric ED, as defined by the decision-making tool PREDAFLU, was a significant parameter. The underlying idea was to provide an early warning of adult ED overload due to CRD by using pediatric data. The pediatric data were extracted by using an easy and already readily available tool.

We also aimed to determine if the occupancy of the ED, percentage of occupancy for CRD diagnosis compared with total occupancy, mean length of ED stay, and ratio of admissions for CRD visits in the adult ED could be discriminating parameters with respect to respiratory infection outbreaks.

## Methods

### Ethics Consideration

This study does not involve intervention on humans, but is an analysis of "emergency room summaries" carried out in accordance with the decrees governing clinical research in France, in particular with regard to the information to be provided to individuals. However, our study started before 2018, the date of promulgation of the decrees, and therefore is not subject to this law [5]. In view of these elements, we have no ethical elements to report according to the French authorities.

The Advisory Committee on Data Processing regarding research in the Field of Health (Comité Consultatif sur le Traitement de l’Information en matière de recherche dans le domaine de la Santé [CCTIRS] Number: 16-660) and the Commission Nationale de l’Informatique et des libertés ([CNIL] Number: DR-2017-394) authorized the collection and the processing of data for this project.

### Study Design

This was an ecological study design based on a retrospective review of data related to admissions to the adult ED. The data processed were raw epidemiologic descriptive data.

CHU Grenoble is located in the heart of Grenoble, a city of about 450,000 inhabitants. In 2019, the adult ED had 59,546 admissions, and the pediatric ED had 33,000 admissions. CHU Saint Etienne is located in an agglomeration of 400,000 inhabitants, and its adult and pediatric EDs registered 53,081 and 35,000 admissions, respectively, in 2019.

Patients were managed similarly within the 2 ED hospital facilities. A triage nurse would start with an evaluation of the patient in order to guide them to the most appropriate emergency area. The care would start in the ED, and then, depending on the clinical state and type of care or investigations required, the patient would be sent home, be transferred to a short stay unit (SSU; Unité d’Hospitalisation de Courte Durée), or be admitted to a medical ward within the hospital. The length of stay in the SSU was different depending on the hospital and the local setting. The decision to direct a patient to the SSU or a standard hospital service was affected by multiple factors including the pathology severity, need for specialized care management, or availability of downstream beds. When a patient was admitted to the SSU, they could either be sent back home after appropriate care or be transferred to another service in the hospital depending on bed availability and type of care required. We can therefore infer that, although the 2 hospitals had similar care pathways, the length of stay in the different services (ED or SSU) could be quite different.

Records for the patient visits to EDs in CHU Saint Etienne and Grenoble (France) from June 29, 2015 to March 22, 2020 were extracted from the Résumés de Passages aux Urgences database (Table 1).

**Table 1:** Population and emergency department (ED) visits from July 2015 to March 2020.

Visit characteristics		During respiratory infection outbreak period	Outside a respiratory outbreak period	All periods
**Total ED visits: CHU^a^ Grenoble**				
	Number of visits	96,870	159,999	256,869
	Mean age (years)	51.54	50.95	51.18
	Men, n (%)	51,922 (53.60)	87,055 (54.41)	138,992 (54.11)
**Total ED visits: CHU Saint Etienne**				
	Number of visits	106,866	139,544	247,410
	Mean age (years)	47.87	47.29	47.55
	Men, n (%)	57,088 (53.42)	75,284 (53.95)	132,909 (53.72)
**ED visits with cardiorespiratory diagnoses: CHU Grenoble**				
	Number of visits	6213	6148	12,361
	Mean age (years)	73.35	75.86	74.9
	Men, n (%)	3284 (52.86)	3460 (56.28)	6744 (54.56)
**ED visits with cardiorespiratory diagnoses: CHU Saint Etienne**				
	Number of visits	6507	6030	12,537
	Mean age (years)	74.34	74.64	74.5
	Men, n (%)	4837 (52.25)	3338 (55.36)	6785 (54.12)

^a^CHU: Centre Hospitalier Universitaire.

### Study Protocol

#### Population

All the admissions to the EDs of CHU Saint Etienne and Grenoble from June 29, 2015 to March 22, 2020 were included in the study. Both hospitals had independent but identical information systems. The 2 hospitals used the International Classification of Diseases (ICD)-10 standard for the classification and standardization of diagnoses. Given the high number of diagnostic codes available, the way a diagnosis was coded in the system could vary from one hospital or one physician to another. Thus, in order to insure the most exhaustive selection among the admissions while obtaining comparable data between the 2 hospitals, we identified all the ICD-10 codes that could be used in cases of CRD diagnosis (Multimedia Appendix 2). This selection was made with the assistance of emergency specialists accustomed to the use of the system, with the objective of having an exhaustive list of diagnostic codes.

The data related to adult ED admissions for CRD diagnosis were thus aggregated by week for the period studied. Admission data linked to ICD-10 codes for CRD were also be compared with the data based on all admissions to the ED, regardless of ICD-10 diagnosis.

**Figure 1:**
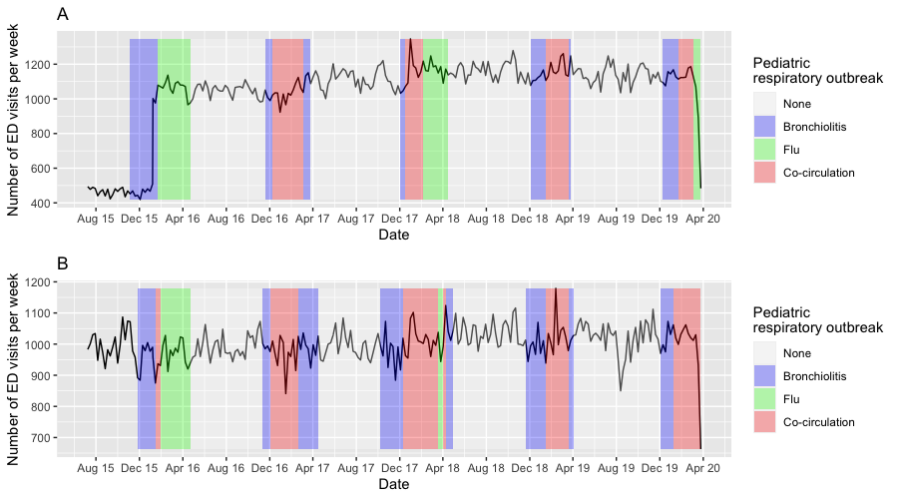
Time series of emergency department (ED) visits for all diagnoses at (A) Centre Hospitalier Universitaire (CHU) Grenoble and (B) CHU Saint Etienne.

**Figure 2:**
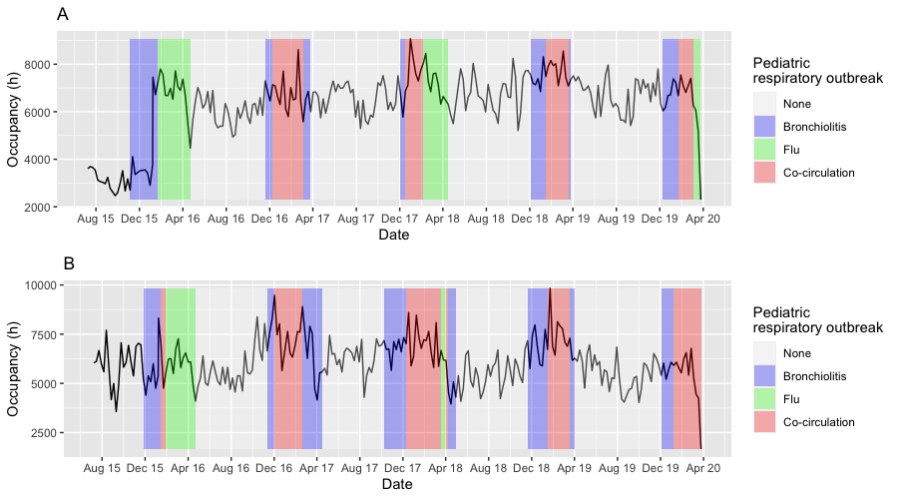
Time series of emergency department (ED) occupancy for all diagnoses at (A) Centre Hospitalier Universitaire (CHU) Grenoble and (B) CHU Saint Etienne.

With regard to the adult population, there was no seasonal increase in activity in terms of weekly number of ED visits in CHU Saint Etienne or Grenoble (Figure 1). Nevertheless, during respiratory pathogen activity, the ED experienced increased workload that could be identified on a time series graph of the occupancy for all ICD-10 diagnoses (Figure 2). In January 2016, the number of visits to the ED of CHU Grenoble nearly doubled compared with 2015. This was due to the merger of 2 EDs in Grenoble. The drop in the number of ED visits or occupancy seen in March 2020 was due to the COVID-19 outbreak.

#### Respiratory Virus–Related Infection Outbreaks in Pediatric EDs

**Figure 3:**
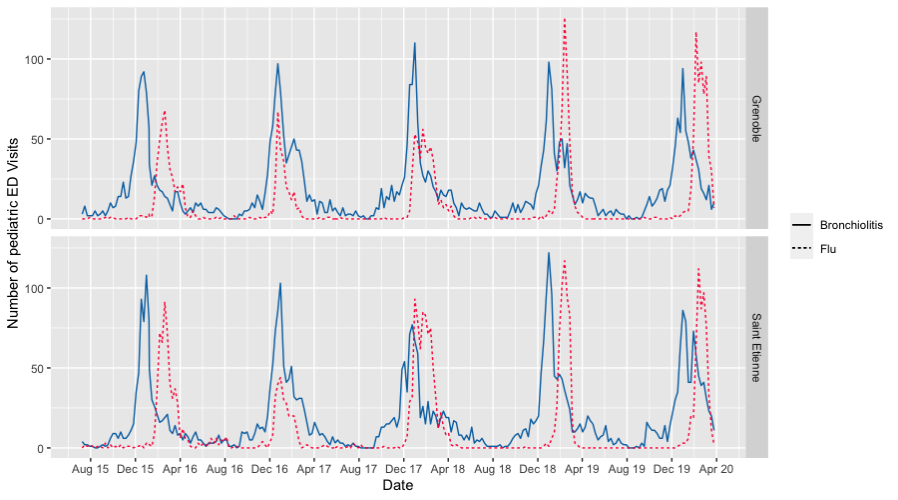
Time series of bronchiolitis and flu outbreaks in the pediatric emergency department (ED) at Centre Hospitalier Universitaire (CHU) Grenoble and CHU Saint Etienne.

The web application PREDAFLU is a web-based decision-making support tool used in pediatric EDs to provide real-time activity monitoring of epidemic episodes of flu and bronchiolitis. The definition of an outbreak period is set in PREDAFLU [6,7] with 50%, 80%, and 100% confidence levels. We decided to define an outbreak period when a 50% confidence was proposed. A flu outbreak was defined as 3 consecutive days of positive tests. A bronchiolitis outbreak was defined as a sudden increase in the number of admissions for this diagnosis, compared with usual trends. Therefore, PREDAFLU allowed a precise definition of the timeframe of epidemic periods between July 2015 and March 2020 (Multimedia Appendix 3). Since the web application took into account the data based on pediatric ED admissions in both CHU Saint Etienne and Grenoble (Figure 3) to define the onset of outbreaks, the dates of outbreaks may vary from one hospital to the other. The advantage of PREDAFLU is that it provides a real-time definition of the periods, whereas the true epidemic periods can only be determined retrospectively. As a consequence, the defined PREDAFLU periods were not true epidemic periods and were larger than the true epidemic periods.

### Statistical Analysis

All analysis was performed on a weekly basis (Monday to Sunday) based on the date of ED attendance. The number of the week was calculated using the ISO 8601 standard. For each week, each hospital center, and all ICD-10 codes, several elements were calculated: the number of ED admissions, ED occupancy, mean length of stay in the ED, percentage of bed occupancy, and percentage of total ED visits.

The nonparametric Kruskal-Wallis variance analysis test was used to determine the differences among the number of ED visits for the identified ICD-10 codes depending on the presence of bronchiolitis or flu outbreaks in the pediatric population. The Spearman correlation test was used to analyze the dependency between the number of adult ED admissions with a diagnosis of CRD and the weekly categorization during or outside epidemic outbreak. The Dunnett test was used to perform a pairwise comparison of the number of ED admissions during each outbreak period (bronchiolitis, flu, or cocirculation) with the number of ED admissions outside the outbreak period. Then, box-whisker plots detailed the results with the median and the 95% CI for each outbreak. Further analysis of variance was conducted to determine if the presence of respiratory infection outbreaks had an impact on other criteria of the health care pathway. The analysis was carried out on the total ED occupancy, percentage of total occupancy, mean duration in the ED, and percentage of total ED visits for the identified ICD-10 diagnostic code. The analysis on these secondary criteria was performed with the same statistical method as for the main criteria.

All analyses were performed using R v3.6.4 (The R Foundation for Statistical Computing).

## Results

Statistical analyses were performed on all the CRD admissions to the adult ED of CHU Saint Etienne and Grenoble from June 29, 2015 to March 22, 2020. The variation of these data, aggregated by weeks, was then analyzed regarding the virus circulation period in the pediatric population.

### CRD ED Visits: Descriptive Analysis of the Time Series

**Figure 4:**
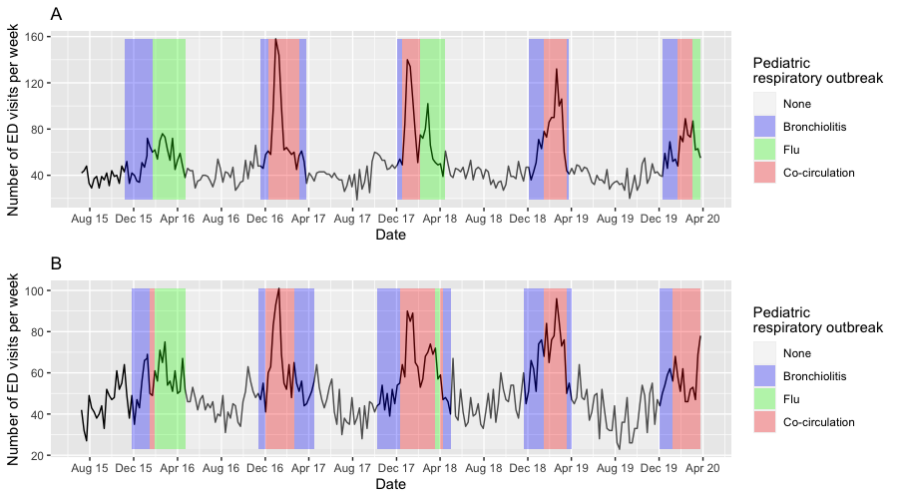
Time series of emergency department (ED) visits for a cardiorespiratory decompensation diagnosis at (A) Centre Hospitalier Universitaire (CHU) Grenoble and (B) CHU Saint Etienne.

For all the identified ICD-10 diagnostic codes, the pattern of the number of weekly visits to the adult ED increased during the outbreak periods of flu and bronchiolitis in the pediatric population for the 5 years studied and in a similar manner at CHU Saint Etienne and CHU Grenoble (Figure 4). During the winter of 2015-2016, the period of cocirculation of bronchiolitis and flu was very short compared with the other years. The peak of the ED visits was lower than the other winters in which the cocirculation period is much more important. For the winters of 2016-2017, 2017-2018, 2018-2019, and 2019-2020, the peak of weekly visits was similar in magnitude and occurred mainly during the cocirculation period.

### Variance Analysis of the Number of ED Visits Related With Respiratory Infection Outbreaks in the Pediatric Population

The analysis of the data for CHU Saint Etienne showed that the median number of ED visits during the outbreak period was 56 (95% CI 54-61) compared with 44 (95% CI 42-46) outside the outbreak periods (ie, a median increase of 12 ED visits per week for CRD pathologies). The difference between these 2 groups, based on the Kruskal-Wallis test, was significant (*χ*^2^_3_=102.7, *P*<.001). The Spearman test showed a moderate positive monotone dependency between the number of ED visits for CRD and the presence of an outbreak as defined by PREDAFLU (*ρ*=0.67; *P*<.001).

The analysis of the data for CHU Grenoble showed that the median number of ED visits during the outbreak period was 60 (95% CI 55-63) compared with 40 (95% CI 38-42) outside the outbreak periods (ie, a median increase of 20 ED visits per week for CRD pathologies). The difference between these 2 groups, based on the Kruskal-Wallis test, was significant (*χ*^2^_3_=126.67, *P*<.001). The Spearman test showed a moderate positive correlation between the number of ED visits for CRD and the presence of an outbreak as defined by PREDAFLU (*ρ*=0.71; *P*<.001).

In addition to the analysis of the link between number of ED visits for CRD and respiratory infection outbreaks in the pediatric population, distinct and separate peaks were observed when breaking down the outbreak periods per respiratory infection type outbreak. We identified 4 different periods. The first period involved no respiratory illness diagnoses in the pediatric ED. The second period corresponded to a bronchiolitis outbreak. The third one was a flu outbreak. And finally, the fourth period was during outbreaks of both bronchiolitis and flu.

**Figure 5:**
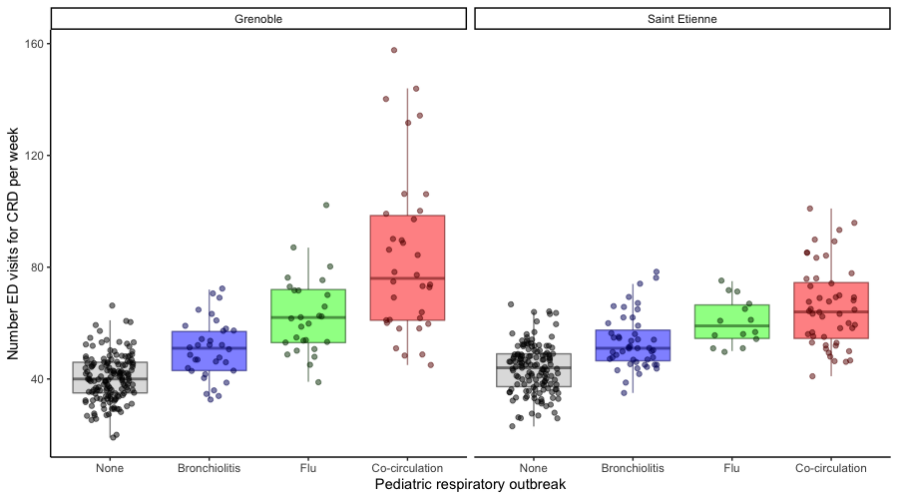
Box-whisker plots of the number of emergency department (ED) visits at (A) Centre Hospitalier Universitaire (CHU) Grenoble and (B) CHU Saint Etienne. CRD: cardiorespiratory decompensation.

The analysis of the data for CHU Saint Etienne showed that the median numbers of ED visits were 51 (95% CI 48-55) during the bronchiolitis outbreak, 59 (95% CI 51-71) during the flu outbreak, and 64 (95% CI 58-69) for flu and bronchiolitis (Figure 5).

The analysis of the data for CHU Grenoble showed that the median numbers of ED visits were 51 (95% CI 46-54) during the bronchiolitis outbreak, 62 (95% CI 53-72) during the flu outbreak, and 76 (95% CI 62-90) for flu and bronchiolitis.

The Dunnett test was used to perform a multiple comparison of the number of ED visits during each outbreak period. The chosen reference for the Dunnett test was the period with no respiratory illness. The Dunnett test was significant for all comparisons (all, *P*<.001). It also highlighted the largest difference for the outbreak period with both bronchiolitis and flu. The results were similar for CHU Saint Etienne and CHU Grenoble (Table 2).

The number of CRD visits to the adult ED of CHU Saint Etienne and Grenoble was a discriminating parameter for populations determined by the nonepidemic and epidemic periods. As a result, the increase in median visits significantly corresponded with the epidemic period defined by PREDAFLU. This increase was also significant for the outbreak of flu alone or bronchiolitis alone but to a lesser extent.

**Table 2:** Dunnett test for multiple comparisons of the number of emergency department (ED) visits for cardiorespiratory decompensation at Centre Hospitalier Universitaire (CHU) Grenoble and Saint Etienne.

Reason for ED visit		Estimated number of visits	SE	*t* value	*P* value
**CHU Grenoble**					
	Bronchiolitis: none	+10.430	2.689	3.878	<.001
	Flu: none	+22.490	2.925	7.689	<.001
	Cocirculation: none	+43.676	2.657	16.440	<.001
**CHU Saint Etienne**					
	Bronchiolitis: none	+9.524	1.745	5.459	<.001
	Flu: none	+16.790	2.897	5.795	<.001
	Cocirculation: none	+22.040	1.731	12.733	<.001

### Results for Secondary Criteria

#### Analysis by Total Occupancy in the ED for a CRD Diagnosis

**Figure 6:**
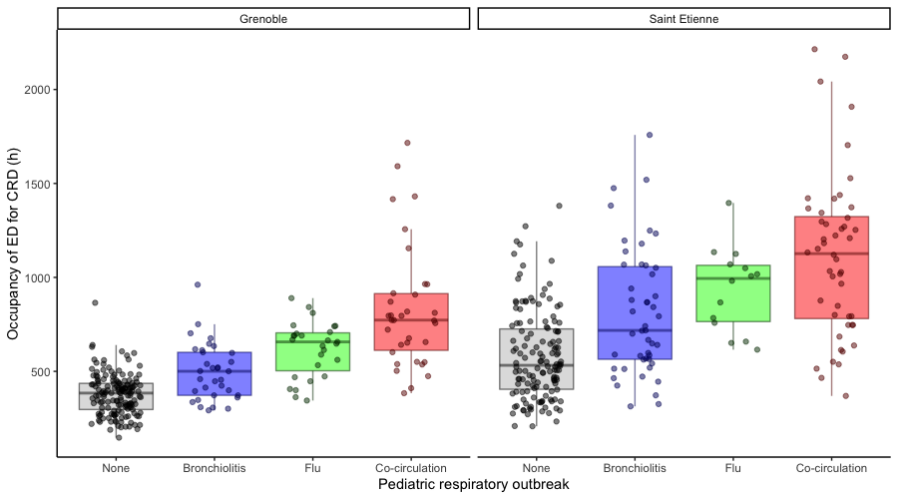
Box-whisker plots of emergency department (ED) occupancy at (A) Centre Hospitalier Universitaire (CHU) Grenoble and (B) CHU Saint Etienne. CRD: cardiorespiratory decompensation.

The total occupancy in the ED for CRD was defined by the total time spent in the ED for all CRD visits during a given week. The difference between the outbreak periods, based on the Kruskal-Wallis test, was significant for CHU Saint-Etienne (*χ*^2^_3_=75.071, *P*<.001) as well as for CHU Grenoble (*χ*^2^_3_=107.12, *P*<.001). The Spearman test showed no real monotone dependency between the occupancy per hour for CRD diagnosis and the presence of an outbreak as defined by PREDAFLU (CHU Saint Etienne: *ρ*=0.55, *P*<.001; CHU Grenoble: *ρ*=0.65, *P*<.001). The monotone dependency was weaker when considering the occupancy compared with the total number of ED visits. The Dunnett tests were significant for all outbreak periods (Table 3). The box-whisker plot for the ED occupancy showed an increase of 69% between the median occupancy outside an outbreak and during an outbreak for CHU Saint Etienne and 63% for CHU Grenoble (Figure 6). The largest increase was during the cocirculation of flu and bronchiolitis (112% for CHU Saint-Etienne; 101% for CHU Grenoble).

**Table 3:** Dunnett test for multiple comparisons of emergency department (ED) occupancy for cardiorespiratory decompensation at Centre Hospitalier Universitaire (CHU) Grenoble and Saint Etienne.

Reason for ED visit		Estimated occupancy	SE	*t* value	*P* value
**CHU Grenoble**					
	Bronchiolitis: none	+121.43	31.73	3.827	<.001
	Flu: none	+242.47	34.50	7.027	<.001
	Cocirculation: none	+451.89	31.34	14.419	<.001
**CHU Saint Etienne**					
	Bronchiolitis: none	+229.11	51.38	4.459	<.001
	Flu: none	+355.26	85.34	4.163	<.001
	Cocirculation: none	+529.17	50.98	10.379	<.001

#### Analysis by Percentage of Total Occupancy

**Figure 7:**
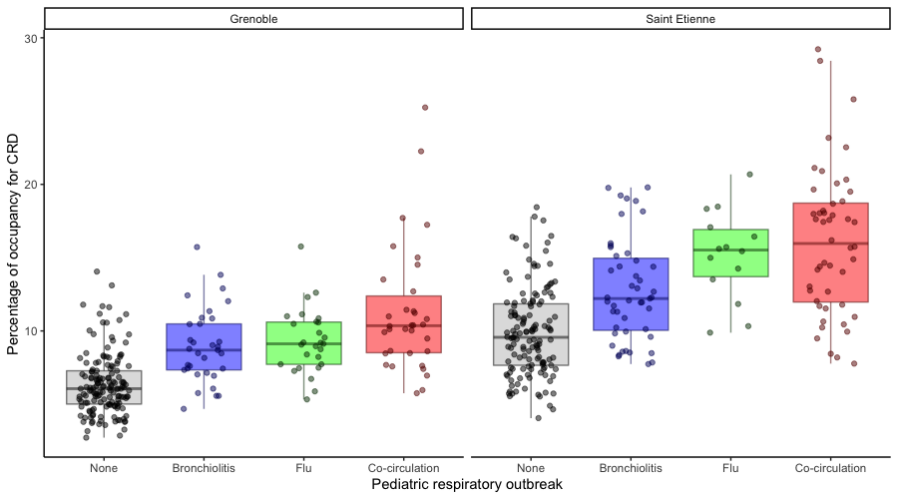
Box-whisker plots of the percentage of emergency department (ED) occupancy for cardiorespiratory decompensation (CRD) at (A) Centre Hospitalier Universitaire (CHU) Grenoble and (B) CHU Saint Etienne.

The percentage of total occupancy was defined by the ratio of total occupancy for CRD to the total occupancy for all diagnoses. The difference between the outbreak periods, based on the Kruskal-Wallis test, was significant for CHU Saint-Etienne (*χ*^2^_3_=77.211, *P*<.001) as well as for CHU Grenoble (*χ*^2^_3_=88.165, *P*<.001). The Spearman test still showed no real monotone dependency between the percentage of occupancy for a CRD diagnosis and the presence of an outbreak as defined by PREDAFLU (CHU Saint Etienne: *ρ*=0.56, *P*<.001; CHU Grenoble: *ρ*=0.60, *P*<.001). The monotone dependency was weaker when considering the occupancy compared with the total number of ED visits. The Dunnett tests were significant for all outbreak periods (Table 4). The box-whisker plot for the percentage of ED occupancy showed an increase of 49% in the percentage of occupancy between the period with no outbreak and with an outbreak for CHU Saint Etienne (Figure 7). The increase was 52% for CHU Grenoble. The largest increase was during the cocirculation of flu and bronchiolitis (67% for CHU Saint-Etienne; 62% for CHU Grenoble).

This result meant the number of ED visits for CRD was more important during an outbreak period, and the workload increase for CRD, measured by the occupancy, was more important during the outbreak relative to the other pathologies.

**Table 4:** Dunnett test for multiple comparisons of the percentage of emergency department (ED) occupancy for cardiorespiratory decompensation at Centre Hospitalier Universitaire (CHU) Grenoble and Saint Etienne.

Reason for ED visit		Estimated percentage	SE	*t* value	*P* value
**CHU Grenoble**					
	Bronchiolitis: none	+2.59	0.4928	5.256	<.001
	Flu: none	+2.94	0.5360	5.487	<.001
	Cocirculation: none	+4.93	0.4868	10.130	<.001
**CHU Saint Etienne**					
	Bronchiolitis: none	+2.87	0.6019	4.771	<.001
	Flu: none	+5.27	0.9996	5.270	<.001
	Cocirculation: none	+6.17	0.5972	10.324	<.001

#### Analysis by Average Duration in the ED for a CRD Diagnosis

**Figure 8:**
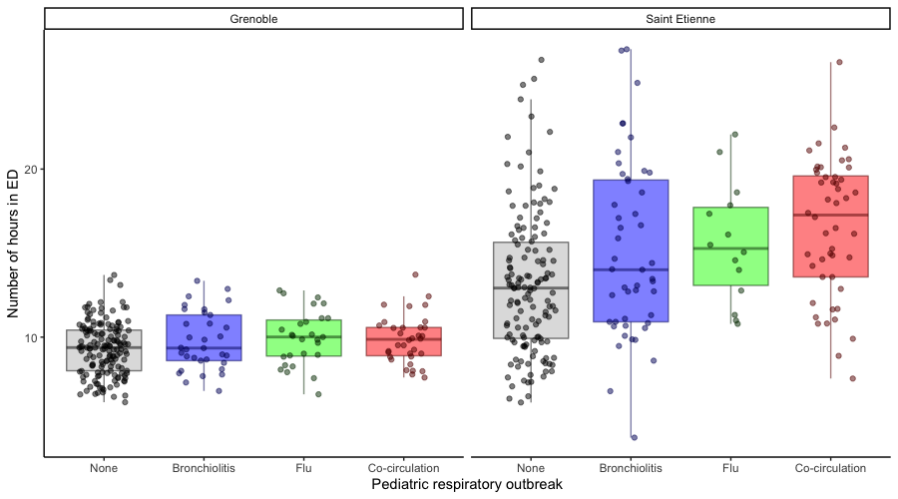
Box-whisker plots of the number of hours in the emergency department (ED) occupancy for cardiorespiratory decompensation (CRD) at (A) Centre Hospitalier Universitaire (CHU) Grenoble and (B) CHU Saint Etienne.

The difference between the outbreak periods, based on the Kruskal-Wallis test, was significant for CHU Saint-Etienne (*χ*^2^_3_=25.495, *P*<.001) but was not significant for CHU Grenoble (*χ*^2^_3_=5.8927, *P*=.12). The Spearman test showed a very weak positive dependency between the mean duration in the ED for a CRD diagnosis and the presence of an outbreak as defined by PREDAFLU (CHU Saint Etienne: *ρ*=0.32, *P*<.001; CHU Grenoble: *ρ*=0.15, *P*=.02). Thus, there was no monotone dependency with the mean duration in the ED for a CRD diagnosis. The Dunnett test was not significant during the flu outbreak at CHU Saint-Etienne and was not significant for all outbreaks at CHU Grenoble (Table 5). Moreover, the box-whisker plot showed a maximum mean increase in the duration in the ED of 4.3 hours at CHU Saint-Etienne and less than 1 hour at CHU Grenoble (Figure 8). This difference was too small to have clinical value.

This result meant the mean duration in ED for CRD diagnosis was independent from the presence or absence of a flu or bronchiolitis outbreak in the pediatric population as defined by PREDAFLU.

**Table 5:** Dunnett test for multiple comparisons of the number of hours in the emergency department (ED) for cardiorespiratory decompensation at Centre Hospitalier Universitaire (CHU) Grenoble and Saint Etienne.

Reason for ED visit		Estimated percentage	SE	*t* value	*P* value
**CHU Grenoble**					
	Bronchiolitis: none	+0.46	0.3012	1.535	0.33
	Flu: none	+0.62	0.3276	1.901	0.16
	Cocirculation: none	+0.55	0.2976	1.836	0.19
**CHU Saint Etienne**					
	Bronchiolitis: none	+1.98	0.7421	2.669	0.02
	Flu: none	+2.39	1.2325	1.943	0.15
	Cocirculation: none	+3.37	0.7363	4.583	<.001

#### Analysis by Percentage of Total ED Visits for CRD

**Figure 9:**
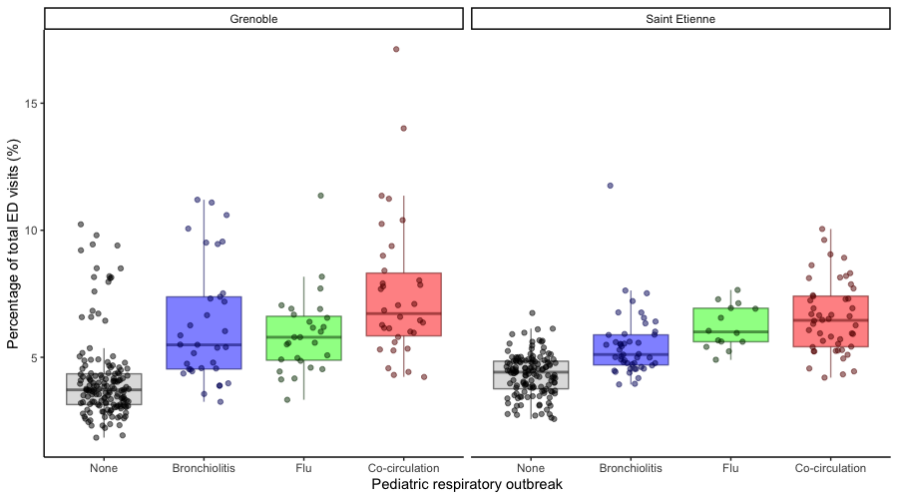
Box-whisker plots of the percentage of total emergency department (ED) visits for cardiorespiratory decompensation (CRD) at (A) Centre Hospitalier Universitaire (CHU) Grenoble and (B) CHU Saint Etienne.

The percentage of total ED visits for CRD was defined as the ratio of the weekly number of ED visits for CRD to the total number of ED visits for all diagnoses. The difference between the outbreak periods, based on the Kruskal-Wallis test, was significant for CHU Saint-Etienne (*χ*^2^_3_=114.5, *P*<.001) as well as for CHU Grenoble (*χ*^2^_3_=93.415, *P*<.001). The Spearman test showed a moderate positive dependency between the percentage of total ED visits for a CRD diagnosis and the presence of an outbreak as defined by PREDAFLU (CHU Saint Etienne: *ρ*=0.68; *P*<.001; CHU Grenoble: *ρ*=0.61, *P*<.001). This was a more significant value. The Dunnett tests were significant for all outbreak periods (Table 6). For this criterion, as for the other, the box-whisker plot for Saint Etienne and Grenoble (Figure 9) showed the largest increase during the cocirculation of flu and bronchiolitis compared with the other outbreak periods (46% for CHU Saint-Etienne; 80% for CHU Grenoble).

**Table 6:** Dunnett test for multiple comparisons of the percentage of total emergency department (ED) visits for cardiorespiratory decompensation at Centre Hospitalier Universitaire (CHU) Grenoble and Saint Etienne.

Reason for ED visit		Estimated percentage	SE	*t* value	*P* value
**CHU Grenoble**					
	Bronchiolitis: none	+2.16	0.3744	5.773	<.001
	Flu: none	+1.77	0.4072	4.342	<.001
	Cocirculation: none	+3.40	0.3699	9.203	<.001
**CHU Saint Etienne**					
	Bronchiolitis: none	+1.15	0.1784	6.438	<.001
	Flu: none	+1.89	0.2964	6.376	<.001
	Cocirculation: none	+2.25	0.1770	12.681	<.001

## Discussion

### Main Findings

The pediatric data concerning the virus circulation were clearly an important variable capable of discriminating between the variations in adult ED visits for CRD. Furthermore, the increase in visits corresponds to an outbreak period. This result was demonstrated for CHU Saint Etienne as much as for CHU Grenoble. The number of ED visits for a CRD diagnosis increased by 27.3% in CHU Saint Etienne and by 50% in CHU Grenoble during a pediatric respiratory infection outbreak. When the data regarding the types of respiratory infections were analyzed, the highest peak was observed during the period of time in which flu and bronchiolitis coexisted. This confirmed the link of an epidemic outbreak to the number of adult ED visits, which was similar for both hospital settings.

The monotone dependency between the number of ED visits for CRD and the presence of a pediatric outbreak as defined by PREDAFLU was moderate but may still be considered as significant. Indeed, the outbreak periods defined by PREDAFLU are based on a real-time analysis of the trends in the pediatric ED for bronchiolitis and flu, with confidence periods of 50%, 80%, and 100% [6,7]. This means the periods defined by PREDAFLU were necessarily larger than epidemic periods classically defined through laboratory-confirmed tests. The moderate dependency found during this study would probably be stronger with a more precise definition of the epidemic periods.

The total ED occupancy for CRD was also a variable capable of significantly discriminating between the populations. During outbreaks of flu or bronchiolitis, we observed an increase in the CRD occupancy by 69.1% in CHU Saint Etienne and by 62.9% in CHU Grenoble. This factor was related to the increased occupancy rate in the ED. The percentage of ED visits for CRD compared with the total number of visits is another variable or parameter of importance, significantly showing the visit evolution during a pediatric respiratory infection outbreak (increase of 28.4% for CHU Saint Etienne and 63.6% for CHU Grenoble).

Finally, the increase in the mean length of stay in the ED was not statically significant nor had clinical significance. Indeed, the increase in the median duration of a stay in the ED for a CRD diagnosis was too small to have a clinical impact on the organization.

This demonstrates that the number of visits for CRD pathologies is more important during outbreak periods relative to the total number of visits, and these visits were also more time-consuming. The combination of these factors highlighted the reason why the increased burden for CRD illness visits has such a strong impact on the adult ED during a pediatric respiratory infection outbreak.

### Strengths and Weaknesses of the Study

The emergency activity related to CRD is not subject to intense seasonal variations. Furthermore, there is no statistical method nor virological rapid tests that allows a precise follow up of such pathologies in the adult ED or can predict increased activity in this setting. This study illustrated the existing relationship between pathologies specific to adult emergency settings and those specific to pediatric emergency settings. One of this study’s main strengths lies in the demonstration of the usefulness of a simple and predictive tool for the pediatric emergency setting like PREDAFLU for an adult setting. This tool has been successfully used for several years in CHU Grenoble and Saint Etienne and allows, through data analysis coming from the pediatric emergency setting, prediction of the start of bronchiolitis and flu outbreaks, with daily updates. PREDAFLU is a simple and reliable tool that could be used to anticipate increased adult ED activity related to CRD.

One weakness of this study concerns the number of hospitals included. Only CHU Grenoble and Saint Etienne took part in the study. They are 2 hospitals of similar sizes and are relatively close geographically. This could constitute a population selection bias.

### Comparison With Prior Work

A key feature of the PREDAFLU surveillance tool is the ability to provide an early warning of respiratory infection outbreaks. This tool is based on syndromic surveillance and on a rapid flu test. The results presented here show that increased ED attendances for CRD may occur during an outbreak of flu or bronchiolitis.

Previous studies have shown the impact of RSV and flu on a higher risk of heart failure [13-16], chronic obstructive pulmonary disease, pneumonia, bronchitis, or other respiratory tract infections [17,18]. Several studies have identified clear predictable patterns driven primarily by viral circulations of respiratory diseases and heart failure within an elderly population [19,20]. Other studies focused on trying to identify leading indicators for the outbreak [21,22], to predict epidemic size by undertaking virologic surveillance [23,24], or to monitor the epidemic periods by performing syndrome surveillance [25-27]. But in all cases, the main challenge is to build a prediction model to forecast an increase in the workload in the ED. Several studies have developed artificial neural network solutions based on environmental, weather, or pollutant data to provide an early detection of peak activities in the ED for respiratory symptoms [28,29]. Other studies used admission information during ED triage to build a prediction model for hospital admission [30]. A review of forecasting applications in health science outlined the challenges in forecasting, including the necessity for a clear definition of health data, the difficulty in forecasting extreme health events, and the necessity to cross-validate the health forecasting models [31]. Other, more general, work has attempted to predict the number of cases presenting to EDs each day. The results are currently rather disappointing. In a fairly focused area, our work contributes to improving the concepts of models that can be used more widely [32].

Our study is unique in that we investigated and showed the benefit of an easy-to-use and reliable forecasting tool for respiratory infections outbreaks in the pediatric ED to anticipate an increase in the activity related to CRD in the adult ED.

### Implications for Clinicians or Managers

The rationale for this work is that epidemics of RSV in the pediatric population may be related to epidemics of CRD in older adults in EDs. This hypothesis assumes that older adults in decompensation are infected with RSV. This has been confirmed in the recent literature, which clearly shows that this virus is found in these clinical situations [33,34] and that it is responsible for a non-negligible fraction of cases [35] on the one hand. On the other hand, this hypothesis implies that RSV circulates in an epidemic mode and that its circulation, when it exists, is massive. This is precisely how RSV epidemics in children are described [36]. Our hypothesis is that RSV, whose circulation increases considerably during bronchiolitis epidemics, is transmitted by children to the elderly and that the circulation of pediatric RSV therefore makes it possible to predict the occurrence of CRD in elderly adults in the ED with better performance than the analysis of adult data alone.

The findings of this study could be useful to emergency physicians and heads of EDs. There could be organizational implications in the use of pediatric emergency indicators related to bronchiolitis and flu outbreaks in order to forecast increased activity due to CRD in the adult population.

The impact of admission for CRD in the adult population has strong implications in the workload of ED and other medicine departments in hospital settings. It usually concerns older populations with significant comorbidities. Furthermore, such pathologies frequently lead to a long hospitalization and require important care organization. The ability to have an indicator to follow (number of CRD visits as shown in this work) such pathologies would allow a much more efficient and effective organization of ED.

A potentially heavy patient overload could indeed be managed in a more flexible way by adapting the ED situation, such as an increase in the number of physicians and nurse shifts or the opening of additional community medicine beds. Hospitals could also communicate more efficiently with medicine departments directly concerned by the increase in such patients (internal, cardiovascular, infectious, or geriatric medicine services). These services would thus be able to forecast sufficient numbers of downstream beds and lighten the burden in emergency services during these difficult periods. The first to benefit from such an organizational shift would be the patients who would then be managed sooner with adequate service.

### Future Work

Admissions to the adult ED used in this study corresponded to previously defined ICD-10 diagnosis, as related to an extended CRD definition. A study of the diagnosis codes used in different hospital settings could lead to the identification of common standard codes that would be more concise and specific.

Furthermore, a study including other seasonal pathologies with virological results from hospital laboratories could possibly help to highlight other relationships between pediatric and adult emergency settings.

Finally, this study would benefit from the inclusion of other hospital settings in different regions of various population size and characteristics, in urban and rural areas. That would allow a validation of this study’s findings on a larger scale and help define a more precise threshold for increased activity in the ED. Bronchiolitis and flu outbreaks can vary in length and intensity; these variations may also be correlated with CRD admissions.

### Conclusion

The analysis of the data concerning ED admissions for CRD in the adult population does not allow for anticipation of the increase in admissions for these diagnoses. This study demonstrated that it is possible to extend the usage of a surveillance tool like PREDAFLU, used to forecast outbreaks of bronchiolitis and flu in the pediatric population, to provide an early warning for an increase in ED visits for CRD in the adult population. This easy-to-use tool can be used to anticipate an overload in ED for these pathologies and to adapt organizational processes.

## Appendix

Multimedia Appendix 1 Time series of cardiorespiratory decompensation (CRD) admission in the adult emergency department (ED) at Centre Hospitalier Universitaire (CHU) Grenoble and Saint Etienne.

Multimedia Appendix 2 Cardiorespiratory selection codes from the ICD-10 Version 2019.

Multimedia Appendix 3 Bronchiolitis outbreak periods.

## Abbreviations

CHU: Centre Hospitalier Universitaire
CRD: cardiorespiratory decompensation
ED: emergency department
ICD: International Classification of Diseases
RSV: respiratory syncytial virus
SSU: short stay unit
