# Supplementary figures and images for: Evaluating the Increased Burden of Cardiorespiratory Illness Visits to Adult Emergency Departments During Flu and Bronchiolitis Outbreaks in the Pediatric Population: Retrospective Multicentric Time Series Analysis
Source: JMIR Public Health Surveill. 2022 Mar 10;8(3):e25532. doi: 10.2196/25532 (PMC8949698; doi:10.2196/25532)

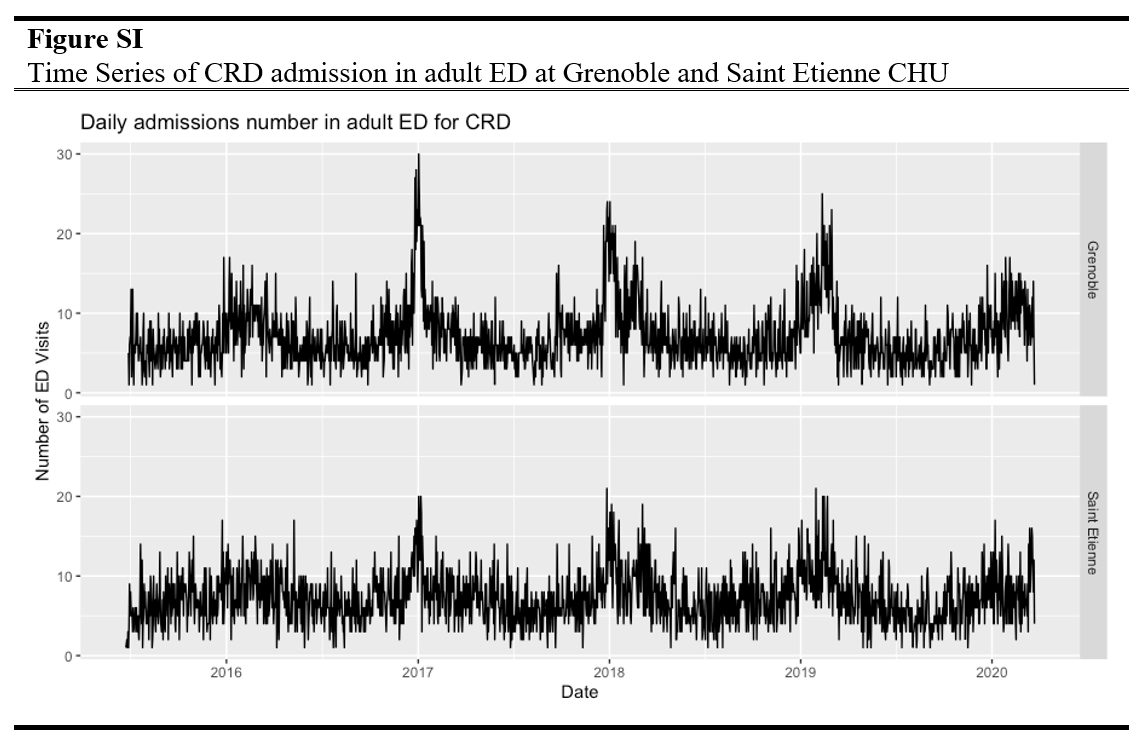

Supplement: Multimedia Appendix 1 [file publichealth_v8i3e25532_app1.png]
