# Supplementary material for: Evaluating the Increased Burden of Cardiorespiratory Illness Visits to Adult Emergency Departments During Flu and Bronchiolitis Outbreaks in the Pediatric Population: Retrospective Multicentric Time Series Analysis
Source: JMIR Public Health Surveill. 2022 Mar 10;8(3):e25532. doi: 10.2196/25532 (PMC8949698; doi:10.2196/25532)
Supplement: Multimedia Appendix 2 [file publichealth_v8i3e25532_app2.docx]

| **Table SI**  Cardiorespiratory selection codes from the ICD-10 Version 2019 | |
| --- | --- |
| Codes | Clinical Diagnoses |
| I50* | Heart failure |
| J09* | Influenza due to identified zoonotic or pandemic influenza virus |
| J10* | Influenza due to identified seasonal influenza virus |
| J11* | Influenza, virus not identified |
| J12* | Viral pneumonia, not elsewhere classified |
| J13* | Pneumonia due to Streptococcus pneumoniae |
| J14* | Pneumonia due to Haemophilus influenzae |
| J15* | Bacterial pneumonia, not elsewhere classified |
| J16* | Pneumonia due to other infectious organisms, not elsewhere classified |
| J17* | Pneumonia in diseases classified elsewhere |
| J18* | Pneumonia, organism unspecified |
| J20* | Acute bronchitis |
| J22* | Unspecified acute lower respiratory infection |
| J40* | Bronchitis, not specified as acute or chronic |
| J41* | Simple and mucopurulent chronic bronchitis |
| J42* | Unspecified chronic bronchitis |
| J44* | Other chronic obstructive pulmonary disease |
| J80* | Adult respiratory distress syndrome |
| J81* | Pulmonary oedema |
| J84* | Other interstitial pulmonary diseases |
| J85* | Abscess of lung and mediastinum |
| J90* | Pleural effusion, not elsewhere classified |
| J96* | Respiratory failure, not elsewhere classified |
