# Supplementary material for: Evaluating the Increased Burden of Cardiorespiratory Illness Visits to Adult Emergency Departments During Flu and Bronchiolitis Outbreaks in the Pediatric Population: Retrospective Multicentric Time Series Analysis
Source: JMIR Public Health Surveill. 2022 Mar 10;8(3):e25532. doi: 10.2196/25532 (PMC8949698; doi:10.2196/25532)
Supplement: Multimedia Appendix 3 [file publichealth_v8i3e25532_app3.docx]

| **Table SII** | | | | | |
| --- | --- | --- | --- | --- | --- |
| Bronchiolitis outbreak periods | | | | | |
|  |  | Start | | End | |
|  |  | Year | Week # | Year | Week # |
| Saint Etienne University Hospital | |  |  |  |  |
|  | Bronchiolitis outbreak periods |  |  |  |  |
|  |  | 2015 | 47 | 2016 | 4 |
|  |  | 2016 | 46 | 2017 | 14 |
|  |  | 2017 | 40 | 2018 | 10 |
|  |  | 2018 | 13 | 2018 | 16 |
|  |  | 2018 | 46 | 2019 | 12 |
|  |  | 2019 | 48 | 2020 | 12 |
|  |  |  |  |  |  |
|  | Flu outbreak periods |  |  |  |  |
|  |  | 2016 | 3 | 2016 | 16 |
|  |  | 2016 | 49 | 2017 | 6 |
|  |  | 2017 | 49 | 2018 | 13 |
|  |  | 2019 | 2 | 2019 | 10 |
|  |  | 2020 | 1 | 2020 | 11 |
|  |  |  |  |  |  |
| Grenoble University Hospital | |  |  |  |  |
|  | Bronchiolitis outbreak periods |  |  |  |  |
|  |  | 2015 | 44 | 2016 | 2 |
|  |  | 2016 | 46 | 2017 | 11 |
|  |  | 2017 | 48 | 2018 | 4 |
|  |  | 2018 | 48 | 2019 | 11 |
|  |  | 2019 | 49 | 2020 | 8 |
|  |  |  |  |  |  |
|  | Flu outbreak periods |  |  |  |  |
|  |  | 2016 | 3 | 2016 | 15 |
|  |  | 2016 | 49 | 2017 | 8 |
|  |  | 2017 | 50 | 2018 | 14 |
|  |  | 2019 | 2 | 2019 | 10 |
|  |  | 2020 | 3 | 2020 | 12 |
